# Erlanger Glaucoma Registry: Effect of a Long-Term Therapy with Statins and Acetyl Salicylic Acid on Glaucoma Conversion and Progression

**DOI:** 10.3390/biology10060538

**Published:** 2021-06-16

**Authors:** Nina Thiermeier, Robert Lämmer, Christian Mardin, Bettina Hohberger

**Affiliations:** 1 Department of Nuclear Medicine, University of Erlangen-Nürnberg, 91054 Erlangen, Germany; nina.thiermeier@uk-erlangen.de; 2 Department of Ophthalmology, University of Erlangen-Nürnberg, 91054 Erlangen, Germany; Robert.laemmer@uk-erlangen.de (R.L.); Christian.mardin@uk-erlangen.de (C.M.)

**Keywords:** ASS, statins, glaucoma, glaucoma suspect, progression, cholesterol

## Abstract

**Simple Summary:**

Glaucoma disease shows a multifactorial pathogenesis, with increased intraocular pressure being the main risk factor. As retinal microcirculation was observed to be impaired in glaucoma, the improvement of capillary blood flow might be an additive option for adjuvant glaucoma therapy. The data of the present study showed that systemic drugs with cardiovascular protective properties (statins, acetylsalicylic acid (ASS)) were observed to have a trend or even significant effect on lowering glaucoma conversion and progression with a time-dependent efficiency. Thus, patients with ocular hypertension and early glaucoma seem to benefit from adjuvant cardiovascular protective therapy, yet potential side effects of systemic therapy with statins and/or ASS should be kept in mind. Thus, a thorough risk–benefit evaluation has to be performed for each patient individually.

**Abstract:**

**Purpose:** Drugs with cardiovascular protective properties (statins, acetylsalicylic acid (ASS)) were assumed to have positive effects on patients suffering from glaucoma disease. The present retrospective study aimed to investigate the influence of statins, ASS or a combination of both on the glaucoma conversion and progression rate in glaucoma suspects and glaucoma patients with a 20-year follow-up period. **Methods:** A retrospective analysis of 199 eyes of 120 patients (63 male, 57 female) of the Erlanger Glaucoma Registry (EGR; ClinicalTrials.gov Identifier: NCT00494923; ISSN 2191-5008, CS-2011) was performed considering systemic therapy with statins, ASS or a combination of both: 107 eyes with ocular hypertension (OHT) and 92 eyes with pre-perimetric primary open-angle glaucoma (pre-POAG). All patients received an ophthalmological examination including morphometric and functional glaucoma diagnostics. Glaucoma conversion was defined as the conversion of OHT to pre-POAG. Glaucoma progression was defined as confirmed visual field loss. Data were shown as percentages. Statistical analysis was performed by Chi-Quadrat tests. **Results:** 1. Glaucoma conversion/progression was observed in 46.7% of the subjects, additionally in combination with hypercholesterinemia in 76.8%. 2. Statins: 27.3% of eyes under systemic statin therapy showed a conversion/progression. Patients taking statins ≥ 10 years yielded a reduced conversion/progression rate (*p* = 0.028, non-significant after Bonferroni–Holm). 3. ASS: 34.7% of eyes under systemic ASS therapy showed a conversion/progression. A significantly lower conversion/progression rate was observed after ASS therapy ≥ 12 years (*p* = 0.017, significant after Bonferroni–Holm). 4. ASS and statins: 25.0% of eyes under combined therapy showed a conversion/progression. A significantly reduced conversion/progression rate was reached after 8 years of combined therapy (*p* = 0.049, non-significant after Bonferroni–Holm). **Conclusions:** Patients with ocular hypertension and early glaucoma seem to benefit from adjuvant cardiovascular protective therapy. However, the benefits and disadvantages of treatment with statins and/or ASS should be kept in mind. Thus, a thorough risk–benefit evaluation has to be performed for each patient individually to avoid unwanted side effects.

## 1. Introduction

Glaucoma is the second leading cause of blindness in the world [1,2]. The prevalence of manifest glaucoma is estimated to be about 1%, increasing with age [3,4]. Thus, glaucoma is expected to have a major impact on economic issues, since healthcare costs will rise with progressive glaucoma disease due to early retirement and patients’ increasing demand of help [5,6]. Glaucoma disease is characterized by a proceeding death of retinal ganglion cells. An increased intraocular pressure (IOP) has been established as a major risk factor in the multifactorial pathogenesis. Additionally, an altered blood flow, genetic alterations, oxidative stress and immune phenomena are involved in this complex interplay [7,8,9,10,11,12,13,14,15].

The presence of cardiovascular risk factors (e.g., diabetes, arterial hypertension, hyperlipidemia) was observed to enhance disease progression in glaucoma patients [16,17,18,19,20,21]. An impaired retinal microcirculation with additional vascular dysregulation might be the basis for this clinical observation. Thus, the improvement of the microcirculation might be a potential target of additional adjuvant therapy [22,23]. It was observed that impaired retinal microcirculation is a very early factor in glaucoma pathogenesis [24,25,26]. A reduction in cardiovascular risk factors with a consecutive improvement in systemic blood flow and thus additional ocular/retinal blood flow may be a benefit for patients with glaucoma [27,28].

The data of a study in 2004 already indicated that the long-term use of cholesterol-lowering drugs may lower the risk of open-angle glaucoma (OAG), especially in those with additional cardiovascular or lipid diseases [29]. Statins are well tolerated cholesterol-lowering drugs, which are beside their cholesterol-lowering effect also known to reduce the risk for cardiovascular and cerebrovascular diseases [30,31]. Systemic therapy with statins due to hyperlipidemia seemed to reduce the risk of glaucoma as well [28,32]. A clinical trial in 2018 (Durham, NC, USA) showed that a decreased visual field progression was observed in patients under actual or past therapy with statins compared to glaucoma patients without systemic use of statins [33].

Therefore, we assume that an improvement in systemic blood flow by a reduction in cardiovascular risk factors might have positive effects on the perfusion of the optic nerve and consequently on glaucoma conversion and progression.

Besides an improvement in systemic blood flow, statins show an additional pleiotropic effect. They upregulate and activate endothelial NO synthase (eNOS) by the inhibition of isoprenoid synthesis. This results in a decrease in Rho GTPase with consequently increased production and bioavailability of endothelium-derived NO [34,35]. These pleiotropic, anti-inflammatory, antiapoptotic and antiproliferative effects were assumed to protect the retinal ganglion cells [36,37].

Next to statins, additional cardioprotective drugs are of interest for the improvement of systemic blood flow (e.g., acetylsalicylic acid, ASS). Acetylsalicylic acid is an unselective cyclooxygenase-1 (COX-1) inhibitor which inhibits platelet aggregation by blocking the formation and release of more than 90% of thromboxane 2 [38]. This anticoagulant effect of ASS might have a positive impact on the perfusion of retinal ganglion cells and the optic nerve as well. To the best of our knowledge, there is no study available in the literature investigating the effect of single ASS therapy and combination with statins on the glaucoma conversion and progression rate with a long-term follow-up.

The aim of the present study was to investigate the effect of systemic therapy with statins and/or ASS on the glaucoma conversion and progression rate in patients with ocular hypertension (OHT) and pre-perimetric primary open-angle glaucoma (pre-POAG) with a follow-up of 20 years.

## 2. Materials and Methods

One hundred ninety-nine eyes of 120 patients of the Erlanger Glaucoma Registry of the Department of Ophthalmology, University of Erlangen (EGR; ClinicalTrials.gov Identifier: NCT00494923; ISSN 2191-5008, CS-2011) were analyzed retrospectively regarding hypercholesterinemia and therapy of single acetylsalicylic acid (administered due to, e.g., coronary heart disease, state after heart attack), single statin (administered due to, e.g., hypercholesterinemia) or combination of both: 107 eyes with ocular hypertension (OHT) and 92 eyes with pre-perimetric primary open-angle glaucoma (pre-POAG). EGR is a longitudinal follow-up study under therapy, starting in 1991. Participants with manifest open-angle glaucoma, glaucoma suspects and a control group are included. Exclusion criteria of participants of EGR are any previous ophthalmic disorder (except glaucoma, OHT) or systemic diseases with ocular affection. Statistical analysis was performed with a follow-up period of 20 years.

All patients underwent a complete ophthalmological examination including slit-lamp biomicroscopy, funduscopy, Goldmann applanation tonometry, standard white-on-white full-field perimetry (Octopus 500, 900 G1 protocol, Interzeag, Schlieren, Switzerland) and Spectralis Optical Coherence Tomography (Spectralis^®^ OCT, Heidelberg Engineering, Heidelberg, Germany).

Glaucoma diagnosis was based on the following criteria:

1. Pre-POAG—open angle of the anterior chamber, intraocular pressure higher than 21 mmHg (repeated twice, Goldmann tonometry), glaucomatous appearance of the optic nerve head, classified after Jonas [39], and normal perimetric findings;
2. OHT—intraocular pressure higher than 21 mmHg (repeated twice, Goldmann tonometry) with normal optic disc appearance and normal perimetry.

Glaucoma conversion was defined as conversion of OHT to pre-POAG. Glaucoma progression was defined as confirmed visual field loss in at least two examinations according to the following criteria:

≥2 adjacent test points, probability of <1%, or;
≥3 test points, probability of <5%, and;
MD > 2.8

The study protocol was performed in accordance with the tenets of the Declaration of Helsinki and was approved by the local ethics committee of the University of Erlangen (3458, 14 February 2006). The statistical analysis was performed using SPSS (version 21.0). The assignment of each participant of EGR to the corresponding analysis group was performed retrospectively according to each individual anamnestic drug report. Data are presented as percentages, mean ± standard deviation. All data were corrected after Bonferroni–Holm considering multiple testing.

## 3. Results

Conversion to glaucoma and glaucoma progression were seen in 69/199 eyes (34.7%) during the follow-up period. Hypercholesterinemia was observed in 87/120 patients (72.5%). Considering hypercholesterinemia as a comorbidity in patients with ocular hypertension (OHT) and pre-perimetric primary open-angle glaucoma (pre-POAG), 76.8% of all patients with glaucoma conversion or progression had elevated cholesterol levels.

**Figure 1:**
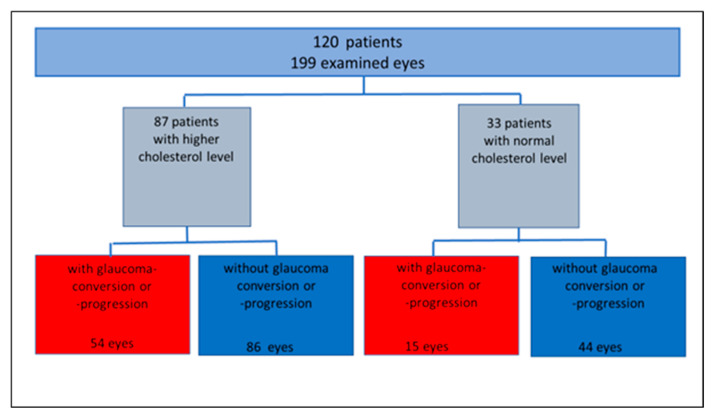
Study population, subgrouped according to cholesterol level, glaucoma conversion and progression rate (absolute number).

Subdividing all eyes into groups of normal (group 1, n = 59 eyes) and elevated cholesterol levels (group 2, n = 140 eyes), 25.4% of group 1 and 38.6% of group 2 showed a glaucoma progression or conversion. A total of 74.6% of group 1 and 61.4% of group 2 yielded stable glaucoma follow-up parameters during the observation period (Figure 1).

### 3.1. Systemic Statin Therapy

The analysis of a potential effect of systemic therapy with statins was performed in 55/199 eyes (27.6%): 15/55 eyes (27.3%) showed a glaucoma conversion or progression. Forty of 55 eyes (72.7%) yielded stable glaucoma parameters during the follow-up period.

**Figure 2:**
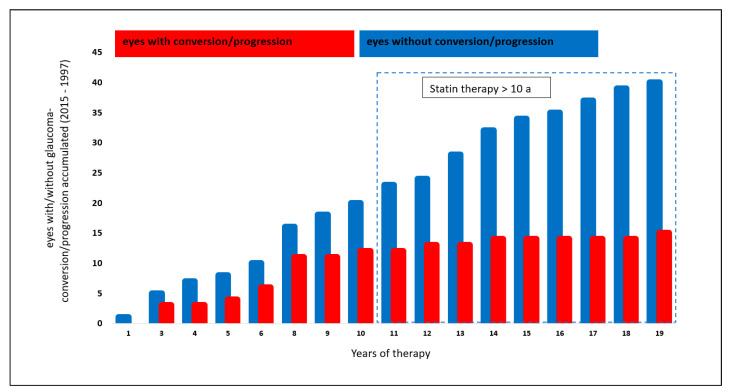
Effect of statin therapy on glaucoma conversion and glaucoma progression.

Analyzing the number of eyes with glaucoma conversion or progression, eyes with systemic statin therapy for a time range of >10 years showed a trend towards a lower conversion and progression rate compared to a statin therapy of ≤10 years (*p* = 0.028, Chi-Quadrat test, non-significant after Bonferroni–Holm; Figure 2).

### 3.2. Systemic Acetylsalicylic Acid (ASS) Therapy

The analysis of systemic ASS therapy included 49/199 eyes (24.6%): 17/49 (34.7%) showed a glaucoma conversion or progression, whereas 32/49 of the eyes (65.3%) did not progress or convert.

**Figure 3:**
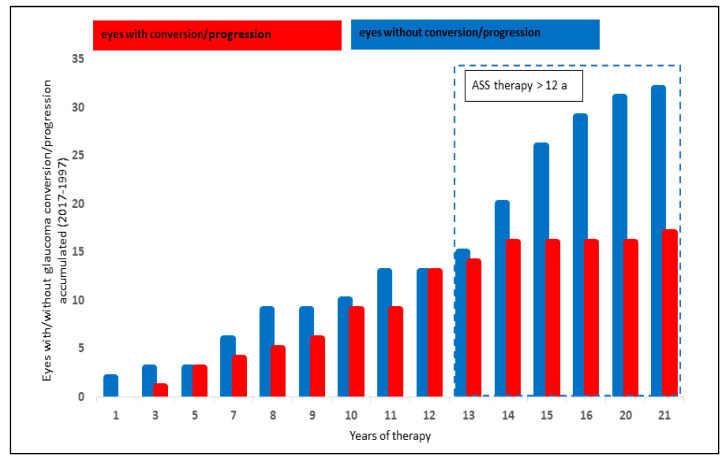
Effect of ASS therapy on glaucoma progression and glaucoma conversion rate.

A significantly lower number of eyes with glaucoma conversion and progression was observed after systemic ASS therapy of ≥ 12 years (*p* = 0.017, Chi-Quadrat test, significant after Bonferroni–Holm; Figure 3).

### 3.3. Combined Systemic Statin and Acetylsalicylic Acid Therapy

The analysis of the effect of systemic therapy with statins and ASS on the glaucoma progression and conversion rate was performed in 28/199 eyes (14.1%): 7/28 eyes (25%) showed a glaucoma conversion or progression, whereas 21/28 eyes (75%) showed stable glaucoma parameters.

**Figure 4:**
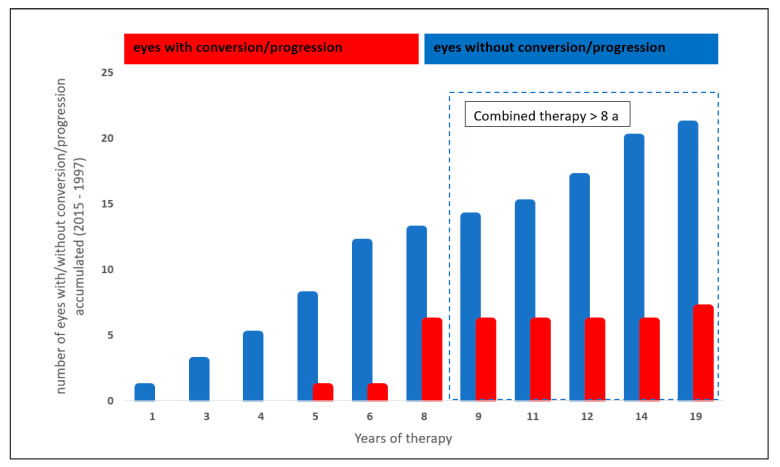
Effect of a combined statin and acetylsalicylic acid therapy on glaucoma conversion and glaucoma progression.

Eyes with a combined systemic therapy of ASS and statins showed a trend towards a lower glaucoma conversion and progression rate after 8 years with the medication (*p* = 0.049, Chi-Quadrat test, non-significant after Bonferroni–Holm; Figure 4).

## 4. Discussion

After 1945, cardiovascular diseases accompanying arteriosclerosis were observed to be one of the main risk factors of mortality, growing rapidly in industrial nations [40]. Still in today’s world, cardiovascular diseases such as ischemic heart disease and stroke are the leading causes of global mortality [41]. Therefore, an effective prevention of cardiovascular diseases is of high importance in the western world [42]. Besides healthy nutrition and physical exercise, drugs (e.g., statins, acetylsalicylic acid (ASS)) were administered to the patients as preventive therapy for cardiovascular diseases [43]. An impaired blood flow [44,45,46,47,48] and vascular dysregulation were observed to play a pathophysiological role in glaucoma disease [11,23,49,50]. Thus, we hypothesize that systemic therapy with ASS and/or statins might be beneficial for patients with glaucoma or a risk of glaucoma. The data of the present study showed that the glaucoma conversion and progression rate showed a trend towards lowering or was even significantly lower when patients were under a systemic long-term therapy of statins, ASS or a combination of both.

Systemic statin therapy for a time range >10 years showed a trend towards a lower glaucoma conversion and progression rate compared to lower intervals. These data conform with the data of Joshua D. Stein et al. [28]. Patients with open-angle glaucoma (OAG) and hyperlipidemia seemed to benefit from statin therapy as the progression rate was observed to be lower. Statin medication of 2 and 5 years reduced the risk of glaucoma by about 8% and 5%, respectively. Even a 40% reduced risk of glaucoma was reported by Mc Gwinn et al. under a statin therapy for >1 year [29]. Perimetric [51] and retinal nerve fiber layer loss [52] was even lower under systemic statin therapy than without. Statins are commonly used to reduce the risk of developing cardiovascular diseases via the improvement of blood flow and so-called pleiotropic effects. As statins inhibit HMG-CoA reductase, being the key enzyme for cholesterol biosynthesis, they majorly reduce the production of the low-density lipoprotein cholesterol. Yet, statins do not only reduce cholesterol biosynthesis via inhibiting HMG-CoA, as they also reduce the production of isoprenoid intermediates (e.g., farnesyl pyrophosphate and geranylgeranyl pyrophosphate) [53] with consecutive decreased isoprenylation of signaling proteins such as Ras, Rho and Rac. The inhibition of RhoA yields an increase in endothelial NO synthase (eNOS) expression, enhancing the production of NO [53]. NO mediates vascular relaxation, reduces platelet aggregation and inhibits leucocyte–endothelial interactions. Statins might improve endothelial dysfunction via an increase in NO [53]. Further, statins are suggested to have anti-inflammatory effects by decreasing the inflammatory cells in arteriosclerotic plaques. It is still elusive if this is related to an inhibition of adhesion molecules such as intercellular adhesion molecule 1 [54]. Statins were also considered to improve the optic nerve’s blood supply via NO-mediated vasodilatation [55]. As statins inhibit HMG-CoA reductase and mevalonate synthesis, they do not only lower cholesterol biosynthesis but also molecules such as isoprenoids. Isoprenoids cause modifications in cell signaling molecules including small GTP binding protein Rho. Rho kinase activation negatively regulates NO production, suppressing NO-mediated vessel dilatation. It is assumed that NO-mediated dilatation triggered by statins appeared by the inhibition of the mevalonate–Rho pathway in retinal arterioles [56]. This hypothesis was supported by Nagaoka et al. [57], showing data that the inhibition of the mevalonate–Rho kinase pathway in endothelial cells plays a part in the simvastatin-induced vasodilation in porcine retinal arterioles. Statins induce NO production in endothelial cells by the phosphorylation of eNOS via PI3-kinase and protein kinase A, independent of HMG-CoA or mevalonate [58].

Each improvement of ocular and systemic microcirculation decelerates glaucoma progression by preventing cell death caused by a reduced blood flow [59]. The effects on blood circulation due to statin therapy were confirmed in animal models (e.g., porcine or bovine retinal cells) [57,58]. In vivo data (rat) proposed a protective effect of statins on retinal ganglion cells’ survival [60]. This neuroprotective effect is probably due to the inhibition of glutamine-induced cytotoxicity by statins [61,62] with a consecutive lower apoptotic rate [63]. In addition, statin therapy seemed to influence intraocular pressure (IOP). These drugs support the drainage of aqueous humor throughout the trabecular meshwork in pig eyes [64]. Lovastatin downregulated the secreted protein acidic and rich in cysteine (SPARC) expression by increasing the expression of Krüppel-like factor 4 (KLF4) [65]. SPARC is a major mediator of aqueous humor outflow by inducing fibrosis and tissue remodeling of the extracellular matrix [66]. As in vivo data showed a reduced IOP (up to 15–20%) in SPARC-null mice, SPARC is assumed to be involved in glaucoma pathogenesis [67,68].

Considering systemic ASS therapy, patients of the present study cohort showed a significantly lower number of glaucoma conversions and progressions after ASS therapy ≥ 12 years. ASS is an unselective inhibitor of cyclooxygenases. A low dose of ASS (100 mg) has an anticoagulant effect by inhibiting the activity of cyclooxygenase 1 (COX-1). The same effect was observed for cyclooxygenase 2 (COX-2) using higher doses [69]. COX 1 usually converts arachidonic acid into prostaglandin H2, being processed to thromboxane (TX), playing an important role in platelet aggregation. ASS can reduce >90% of thromboxan-A2 (TX-A2) production [38], thus ASS can improve blood flow via inhibiting platelet aggregation. The inhibition of prostaglandin synthesis in turn stimulates the production of prostaglandin receptors in retinal microcirculation [70]. Interestingly, an examination of donor human eyes with different forms of glaucoma showed a reduced number of prostaglandin receptors (especially COX-2) in primary open-angle glaucoma (POAG) [71]. As prostacyclin and prostaglandin E2 act as vasodilators in retinal and choroidal circulation, as seen in rat eyes, a higher amount of prostanoid IP and EP2 receptors might also play an important role in the regulation of ocular hemodynamics [72]. These data supported the hypothesis that ASS causes an increase in prostaglandin receptors in retinal vessels with consecutive improved microcirculation. Considering the concept of an impaired retinal microcirculation in glaucomatous eyes, the systemic benefit of ASS therapy might also be present in ocular, especially retinal, tissue [70,71,73]. Kaplan–Meier curves yielded a higher cumulative probability of glaucoma progression in patients without than with systemic antiplatelet and anticoagulant medication [74].

Patients treated with a combined therapy of statins and ASS showed a trend towards a lower glaucoma conversion and progression rate after >8 years of drug intake. This effect was observed for other diseases as well. Patients showed a better long-term outcome after percutaneous coronary intervention (PCI) by altering both the inflammatory process and the progression of arteriosclerosis [75].

The present study is not without limitations. As the study design was retrospective, we cannot present placebo-controlled study data. Furthermore, additional studies are necessary with increased study cohorts in different countries in order to see if lifestyle and a difference in nutrition, both with an impact on microcirculation, do have an additional effect. On top of that, it is well known that there is a relationship between increasing age [76,77] and the frequency of glaucoma disease as well as between age and increased vascular issues [78].

The latter one is associated with an increased use of cardiovascular protective drugs such as statins and ASS [79]. It could be difficult to define whether the possible interactions between both might affect the present study results. Another point to discuss is adverse events of statins and ASS when those drugs are considered to be used as an adjuvant glaucoma therapy. Diabetes mellitus, myopathy, rhabdomyolysis and hepatotoxicity were reported as potential side effects of statin therapy [79]. An increased risk of bleedings (e.g., gastrointestinal bleeding, subarachnoid hemorrhage) goes along with ASS administration [80]. In addition, the risk of bleeding increases with age, drug–drug interactions or pre-existing gastrointestinal ulcers [81]. On top of that, the discontinuous use of low-dose ASS therapy is suspected to increase the risk for ischemic events such as stroke and TIA in patients with cardiovascular diseases [82]. Risk and benefits should be addressed individually in order to guarantee a positive benefit-to-risk ratio [83]. Otherwise, adverse events may outweigh the benefit. The exact mechanism of action of statins/ASS on glaucoma is not yet thoroughly known. In order to elucidate the precise mode of action, additional studies are required.

## 5. Conclusions

Statins and acetylsalicylic acid (ASS) are well known drugs with benefits for patients suffering from cardiovascular diseases [84,85]. Systemic medication with statins and/or ASS was observed to have a trend or even a significant lowering effect on glaucoma conversion and progression with a time-dependent efficiency. Thus, patients with ocular hypertension and early glaucoma seem to benefit from adjuvant cardiovascular protective therapy. As ASS therapy is associated with a higher risk of gastrointestinal bleeding and ischemic events, being a potential life-threatening side event, systemic therapy should be considered carefully [83,86]. However, the benefits and disadvantages of treatment with statins and/or ASS should be kept in mind. A thorough risk–benefit evaluation has to be performed for each patient individually to avoid unwanted side effects.

## Data Availability

Data are contained within the article. The data presented in this study are available in the Erlanger Glaucoma Registry of the Department of Ophthalmology, University of Erlangen (EGR; ClinicalTrials.gov Identifier: NCT00494923; ISSN 2191-5008, CS-2011).
